# A Russian Dolls ordering of the Hadamard basis for compressive single-pixel imaging

**DOI:** 10.1038/s41598-017-03725-6

**Published:** 2017-06-14

**Authors:** Ming-Jie Sun, Ling-Tong Meng, Matthew P. Edgar, Miles J. Padgett, Neal Radwell

**Affiliations:** 1 Department of Opto-electronic Engineering, Beihang University, Beijing, 100191 China; 2 SUPA, School of Physics and Astronomy, University of Glasgow, Glasgow, G12 8QQ UK

## Abstract

Single-pixel imaging is an alternate imaging technique particularly well-suited to imaging modalities such as hyper-spectral imaging, depth mapping, 3D profiling. However, the single-pixel technique requires sequential measurements resulting in a trade-off between spatial resolution and acquisition time, limiting real-time video applications to relatively low resolutions. Compressed sensing techniques can be used to improve this trade-off. However, in this low resolution regime, conventional compressed sensing techniques have limited impact due to lack of sparsity in the datasets. Here we present an alternative compressed sensing method in which we optimize the measurement order of the Hadamard basis, such that at discretized increments we obtain complete sampling for different spatial resolutions. In addition, this method uses deterministic acquisition, rather than the randomized sampling used in conventional compressed sensing. This so-called ‘Russian Dolls’ ordering also benefits from minimal computational overhead for image reconstruction. We find that this compressive approach performs as well as other compressive sensing techniques with greatly simplified post processing, resulting in significantly faster image reconstruction. Therefore, the proposed method may be useful for single-pixel imaging in the low resolution, high-frame rate regime, or video-rate acquisition.

## Introduction

Imaging is one of the most ubiquitous and useful techniques for gathering information. Imaging is conventionally performed using cameras based on detector arrays and though a very mature technology, these have their limitations. Recently there has been a push towards imaging with only a single detector^1, 2^ and this so-called ‘single-pixel imaging’, also closely related to classical ghost imaging^3, 4^. Rather than capturing a two-dimensional (2D) image with a pixelated array, these techniques use an alternative strategy to retrieve spatial information by recording only the total light intensities in each component of a spatial sampling basis. These intensities corresponding to each of the basis components are measured on a single-pixel detector sequentially in time, and together with knowledge of the sampling basis, an image can then be reconstructed. Though detector array technology has superior performance in the visible region of the spectrum, single-pixel imaging is particularly well-suited to non-conventional imaging, such as multi-wavelength imaging^5^, depth mapping^6–9^, 3D profiling^10, 11^.

The most mature method of single-pixel imaging is the raster scanning approach^12, 13^, where the object is scanned one image pixel at a time. Entering the new century, single-pixel imaging utilized pseudo-thermal random speckle patterns to sample a scene^14, 15^. Advances in computational ghost imaging led to the use of a spatial light modulator (SLM) to generate the random patterns^3, 4^. However, the non-orthogonality of random patterns often means that more than *N* measurements are required for a high quality reconstruction of an *N* pixel image^16^. Improvements can be made by sampling a scene with patterns forming an orthogonal basis set, allowing, in principle, a perfect reconstruction of an *N* pixel image with *N* measurements^17, 18^.

The single frame acquisition time of single-pixel imaging is typically longer than that of a conventional camera due to the need for sequential measurements. Acquisition time can be shortened by reducing the number of measurements, however, this potentially leads to loss of information. Compressed sensing can be used to produce higher quality image reconstructions from fewer than *N* measurements by exploiting the sparsity in the spatial frequencies present in natural scenes. This ‘conventional compressive sensing’^1, 2, 19^, is usually performed by minimizing a certain measure of the sparsity. It is widely understood that the number of measurements required to form a ‘good’ reconstruction is related to the sparsity of the image^2, 20^:

1 $$M/N={\mathscr{O}}(\alpha \,\mathrm{log}\,(1/\alpha )),$$

where *M* is the number of measurements required to form a good reconstruction, *N* is the total pixel number, and *α* is the sparsity ratio of image expressed in the chosen basis. In practice, $M\approx 4\alpha \,\mathrm{log}\,(1/\alpha )N$ is often an adequate number of measurements for good reconstruction, and the dependence of reconstruction quality on M/N can be predicted quite precisely^21^. In this work we do not consider images that have an exact sparse representation^21, 22^, but rather *α* represents the proportion of coefficients greater than some threshold. The ratio *M*/*N* can be considered as a sampling ratio, and for sparse images, improves as *α* decreases. The relationship between *α* and *N* depends on both the scene and the chosen definition of a non-sparse component, however, in general *α* decreases for larger *N*, or rephrased: the larger the dimensionality of the image, the sparser it becomes (in the spatial frequency basis). The overall result is that the sampling ratio scales very favourably with larger pixel numbers and therefore conventional compressed sensing excels at reconstructions of large (megapixel) images, especially when wavelet bases are used^23^. However, for more modest resolutions, one does not obtain good performance when the sampling ratio is below about 30%^24^. Real-time video applications in the 10–30 Hz regime have resolutions typically between 32 × 32 and 128 × 128, limited by the modulation rate (22 Khz) of even the fastest SLM devices^5, 9, 18, 25^. These applications therefore have relatively high *α* and hence conventional compressed sensing technique are often only marginally effective while also incurring long reconstruction times, again, unsuitable for real-time imaging. Therefore alternative compressive approaches have been explored for single-pixel video, such as evolutionary compressive sensing (ECS)^5, 11, 25^, where the measured patterns are chosen based upon *a priori* knowledge of the scene, taken from the previous frame and requires no lengthy post-processing. ECS can achieve real-time imaging but incurs a trade-off between image quality and real-time robustness^25^.

Here we present an alternative approach which can utilize the sparsity in general scenes while avoiding the need for a time-consuming computational overhead and relies on a basic presumption that general scenes are sparse. Our approach is based on an optimized ordering of the Hadamard basis which we call the ‘Russian Dolls’ order, where the reshaped basis patterns are ordered corresponding to their significance for general scenes. We numerically compare the reconstructed images obtained using this ‘Russian Dolls’ order against both a standard conventional compressive sensing technique and evolutionary compressive sensing. We find that for modest resolutions this method can produce similar or better image quality when compared to conventional or evolutionary compressive sensing.

## Principles of image reconstruction

In single-pixel imaging, the measured intensity *S*_*i*_, associated with each measured pattern *P*_*i*_, is directly proportional to the overlap between the pixelated scene *I*_*o*_ and the pattern *P*_*i*_ and a reconstructed image *I*_*r*_ can be obtained using the knowledge of *S*_*i*_ and *P*_*i*_^3, 4^. If the patterns form an orthonormal basis, then an *N* pixelated scene can be fully sampled after performing *N* pattern projections and measurements, and the reconstructed image *I*_*r*_ can be obtained using

2 $${I}_{r}=\sum _{i=1}^{N}\,{S}_{i}\cdot {P}_{i},$$

One such orthonormal basis is derived from the Hadamard matrix; a square matrix with elements ±1 whose rows (or columns) are orthogonal to one another^26, 27^. Each pattern is formed by reshaping a row (or a column) of the Hadamard matrix into a two-dimensional square array. The lowest-order Hadamard matrix is of order two:

3 $${H}_{2}=[\begin{array}{cc}1 & 1\\ 1 & -1\end{array}].$$

Higher order Hadamard matrices are obtained by ${H}_{{2}^{n+1}}={H}_{{2}^{n}}\otimes {H}_{2}$, where $\otimes$ is the Kronecker product operator. The *i* th row/column in the Hadamard matrix can be reshaped to form a square pattern *P*_*i*_. These mathematical operations lead to an ordering of the rows/columns (we will mention rows only hereafter because *H* = *H* ^*T*^), which we call the ‘Natural Order’.

**Figure 1:**
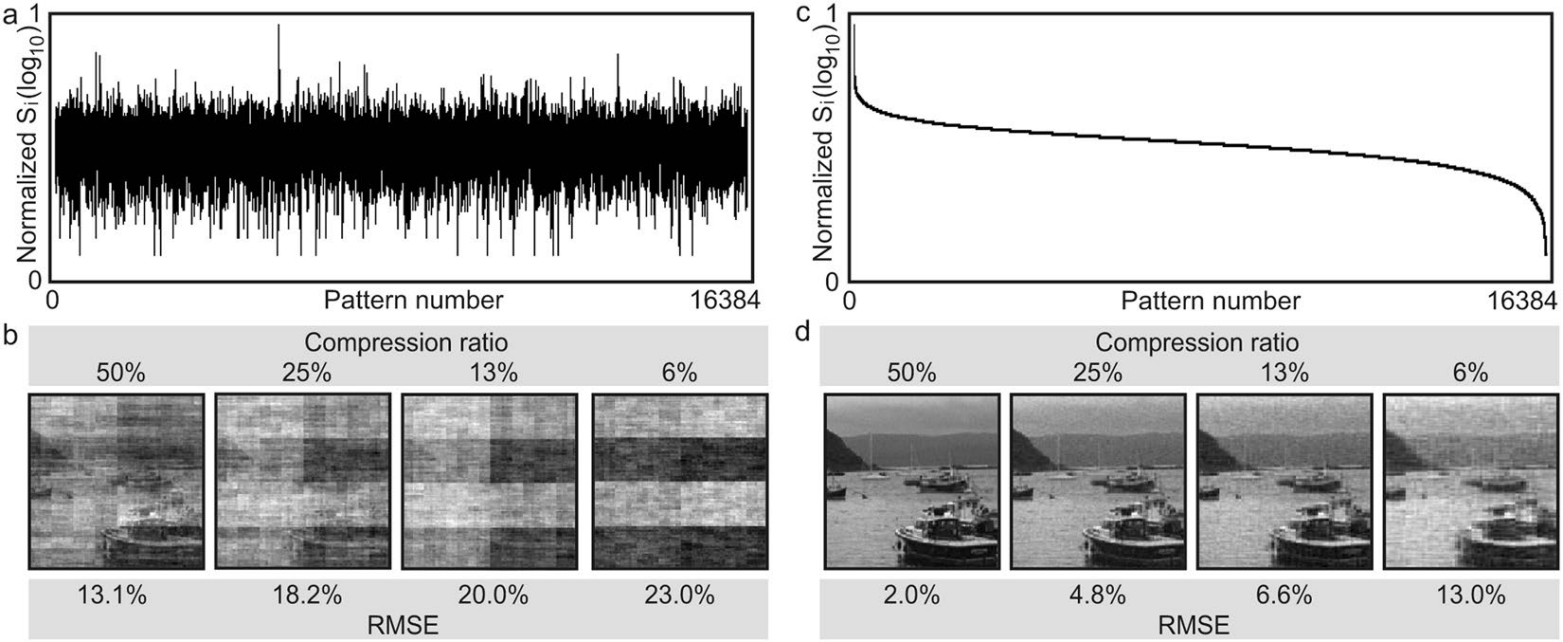
Image reconstruction with different fractions of the complete set of Hadamard patterns. (**a**) Decomposed intensities of 16384 randomly ordered Hadamard patterns. (**b**) Comparison of reconstructions using fractions of the complete set. (**c**) Intensities in descending order. (**d**) Reconstructions using the most significant fractions of the complete set.

Figure 1a shows the calculated intensities (*I*_*S*_) corresponding to measurements of 16384 Hadamard patterns (*P*_*i*_) in a random order, measured from a sample picture. Figure 1b shows the reconstructed images (128 × 128 pixels) when using only a fraction *C* of the complete set, using the first *N* × *C* rows of the Hadamard matrix. The quality of reconstruction is evaluated using the percentage root mean squared error (RMSE), which is calculated by

4 $${E}_{RMS}=\sqrt{\frac{{\sum }_{i,j=1}^{m,n}{({I}_{r}(i,j)-{I}_{o}(i,j))}^{2}}{N}},$$

where *I*_*r*_(*i*, *j*) and *I*_*o*_(*i*, *j*) are the values of the (*i*, *j*) th pixel in the reconstructed and original images respectively, *m* and *n* are the dimensions of the image, and *N* = *m* × *n* is the number of pixels. All images are normalized to unity.

It is a sensible assumption that the larger the signal (*S*_*i*_), the more significant the pattern’s (*P*_*i*_) contribution to the image reconstruction and in order to reduce the number of patterns used, it would be ideal if the most significant patterns are always projected and measured first, this is the fundamental idea of ECS^5, 25^. Figure 1c shows the intensities in their descending order and Fig. 1d shows the reconstructed images using the most significant fractions. The resulting images as well as the relative errors demonstrate that with the same sampling ratio, a significance-based ordering of the Hadamard basis provides a better reconstruction from fewer measurements than a random ordering. ECS, however, has a major drawback as one cannot know which patterns will produce the most significant signals *a priori* and therefore these patterns must be chosen by random sampling from frame-to-frame leading to reconstruction errors for quickly moving scenes.

### Optimising the Hadamard basis order

The Hadamard matrices are common means in multiplexed imaging, though maybe not the best one^28^. In this work, we demonstrate an optimized ordering of the Hadamard basis, where we use the properties of general scenes to order the patterns such that any truncation of that pattern sequence will provide an optimal reconstruction. The rules to order the Hadamard basis patterns are as follows.

#### Rule 1: Order the rows such that the top half of ${H}_{{2}^{2n}}$ are the rows of ${H}_{{2}^{2n-1}}$

This basic principle develops from the realisation that each Hadamard matrix contains within it each lower order Hadamard matrix, for example an *H*_8_ Hadamard matrix contains the rows of a *H*_4_ Hadamard matrix (scaled by a factor 2), which in turn contains the *H*_2_ etc, just like a Russian dolls set. More concisely this can be expressed as ${H}_{{2}^{2n}}$ containing a scaled version of ${H}_{{2}^{2n-1}}$. From this realisation, along with the fact that using a complete Hadamard basis to reconstruct an image provides better signal-to-noise ratio (SNR)^17, 18, 29^, we reorder a ${H}_{{2}^{2n}}$ Hadamard matrix such that the first half rows are ${H}_{{2}^{2n-1}}$, the first quarter rows are ${H}_{{2}^{2n-2}}$, the first eighth rows are ${H}_{{2}^{2n-3}}$ and so on.

#### Rule 2: Ordering the third quarter of ${H}_{{2}^{2n}}$ as the transpose of its second quarter

Following Rule 1, the rows in the first quarter and the second quarter of ${H}_{{2}^{2n}}$ are fixed. According to the symmetry of the Hadamard matrix, the transpose of the second quarter basis patterns can always be found in the latter half of ${H}_{{2}^{2n}}$. Therefore we order the third quarter basis patterns as the transpose of the second quarter of ${H}_{{2}^{2n}}$. Note that the second and third quarters are interchangeable, and with the first quarter, both can form the complete Hadamard basis of ${H}_{{2}^{2n-1}}$.

#### Rule 3: Ordering the patterns within each quarter according to the number of blocks they contain

**Figure 2:**
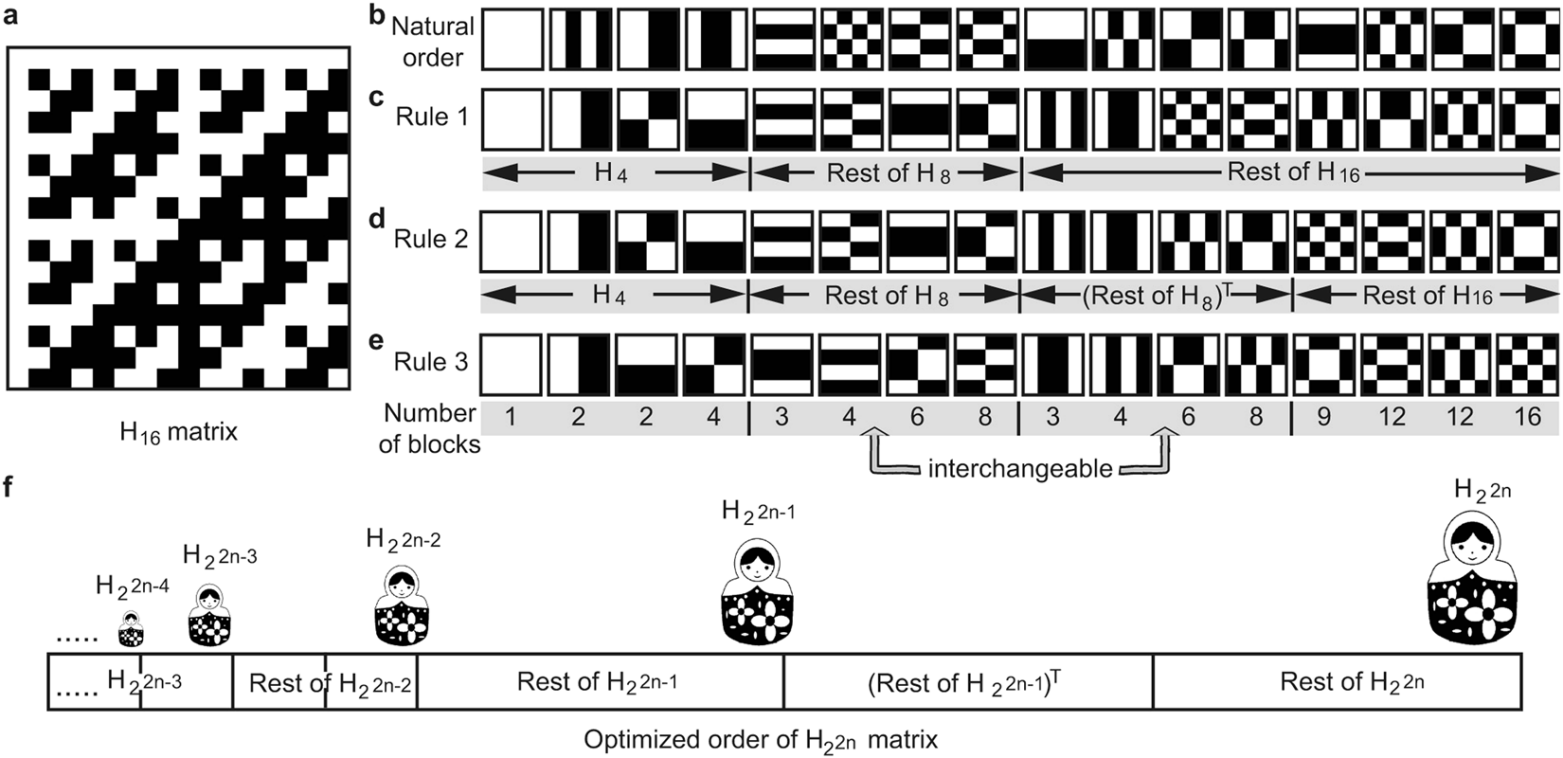
‘Russian Dolls’ Hadamard ordering example. (**a**) A 16 × 16 Hadamard matrix. (**b**) The basis patterns of *H*_16_. (**c**–**e**) The basis patterns of *H*_16_ at different stages during the optimized ordering. (**f**) An example of optimized order of a Hadamard matrix, forming a ‘Russian Dolls’ structure.

Following Rule 1 & 2, all basis patterns are catalogued into the four quarters of ${H}_{{2}^{2n}}$. One value is then given to each reshaped basis pattern, representing the number of blocks it contains. We define a block as an unbroken area of equal value (black or white in Fig. 2). We hypothesize that the less blocks a pattern contains, the more probable this pattern yields a higher intensity signal for a general scene. Therefore, we order the basis patterns within each quarter ascending according to their block number.

Figure 2 gives the example of ordering a 16 × 16 Hadamard matrix using the above rules. By taking each row of the *H*_16_ matrix (Fig. 2a) and transforming each row into a 4 × 4 2D pattern, a complete set of 16 Hadamard basis patterns (Fig. 2b) is obtained, which can be used in single-pixel imaging to reconstruct 4 × 4 resolution images. Following Rule 1, the first half of the patterns are those from the *H*_8_ matrix and the first quarter of the patterns are that from the *H*_4_ (Fig. 2c). We then choose the transpose of the second quarter patterns from the latter half and arrange them into the third quarter (Fig. 2d). Finally, we sort each quarter of the patterns according to their block number (Fig. 2e).

## Results

In order to test our method for image reconstruction, numerical calculations are performed where a set of images *I*_*o*_ are sampled by patterns *P*_*i*_ to yield signals *S*_*i*_ and images *I* are then reconstructed using three different approaches; ‘Russian Dolls’ ordering, evolutionary compressive sensing and conventional compressive sensing. The original images, sampling patterns and resulting images all have resolutions of 128 × 128 pixels. The Hadamard matrix is ${H}_{{2}^{14}}$, with dimension 16384 × 16384. The numerical calculations are performed at sampling ratios set from 1% to 99% at 1% intervals based on a full pattern set of 16384.

With the ‘Russian Dolls’ approach, the resulting images are reconstructed by using the sub-set of the ‘Russian Dolls’ ordering of the Hadamard patterns along with Eq. 1. The evolutionary compressive sensing is performed optimally, i.e. all patterns are measured and ordered corresponding to their *S*_*i*_ values (as in Fig. 1c), and each compressive sub-set is taken from the highest *S*_*i*_ values. For conventional compressive sensing, we randomise the Hadamard patterns using democratisation and recover the image using a sparsity optimisation by minimisation of the total image curvature^5^. The RMSE of the resulting images are computed using Eq. 4. All simulations are performed on a laptop with 2.60 GHz quad core processor and 8.00 GB random access memory (RAM).

**Figure 3:**
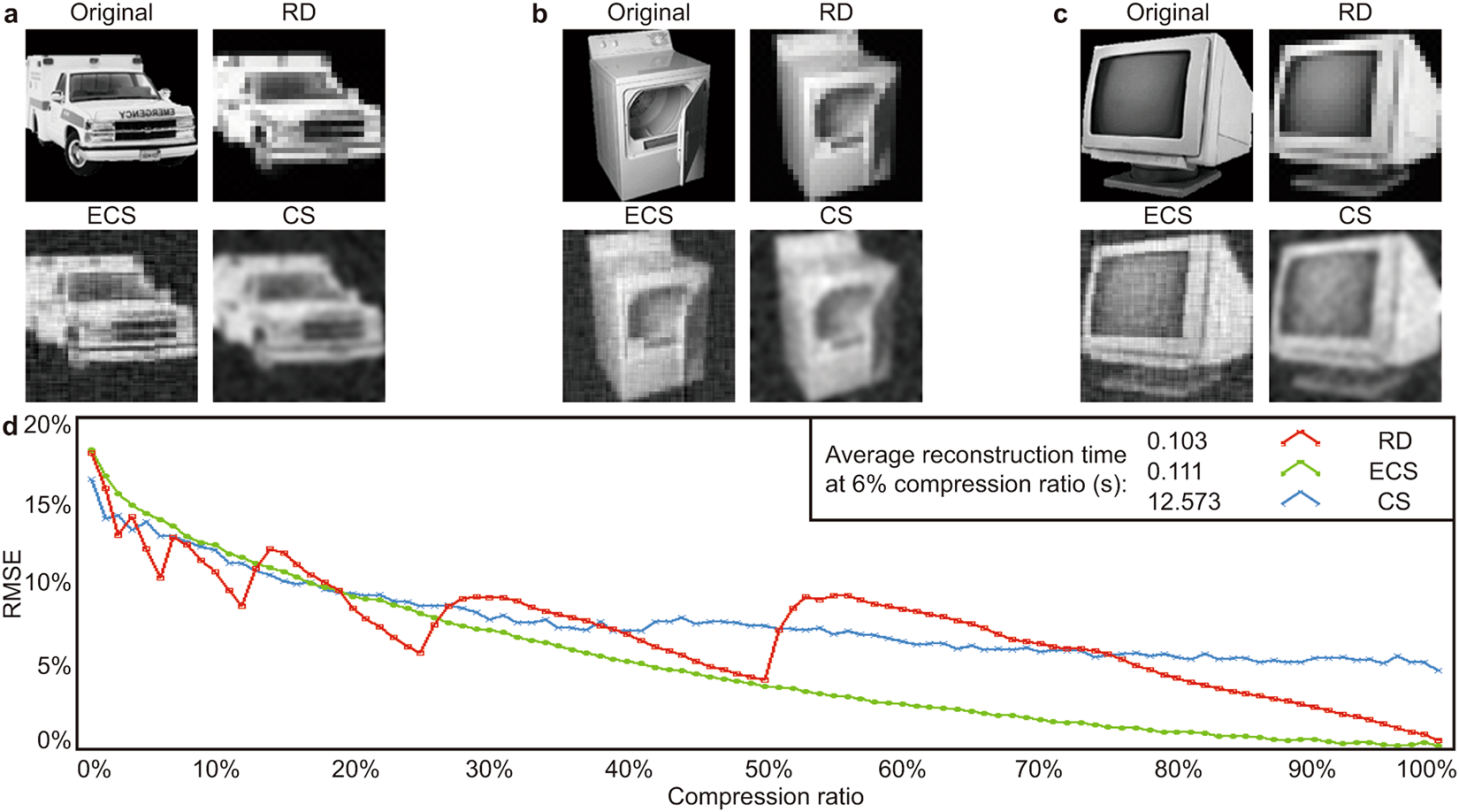
Single object reconstruction comparison. (**a**–**c**) Examples of single objects (Original), the reconstructed images using ‘Russian Dolls’ approach (RD), evolutionary compressive sensing (ECS), and conventional compressive sensing (CS). (**d**) Comparison based on relative error per pixel as a function of sampling ratio. Average reconstruction times at 6% sampling ratio for three approadches are given as well.

In the first simulation, we reconstruct a set of 35 images (three examples of which are labelled as ‘Original’ in Fig. 3a–c), in which each image contains an object on a black background. The reconstructed images at a sampling ratio of 6% are also shown and labelled correspondingly in Fig. 3a–c. Figure 3d shows the RMSE of the reconstruction image as a function of sampling ratio, where the RMSE is the average derived from all 35 reconstructed images.

As expected all three approaches show a similar trend in that the reconstruction quality is improved as the number of patterns increases. For sampling ratios below 20% ECS and conventional compressed sensing perform very similarly, while the ‘Russian Dolls’ ordering result is characterized by some optimal points at 50%, 25%, 12%, 6% and 3% sampling ratio, which is coincident with our prediction that we can form an optimised reconstruction with lower resolution using a sub-set of patterns. For moderate sampling ratios from 20–50% all methods perform very similarly. For sampling ratios above 50% ECS outperforming the other methods. At sampling ratio 6%, the average reconstruction times of all 35 images are 0.103 s, 0.111 s and 12.573 s for ‘Russian Dolls’, ECS and conventional compressive sensing respectively, where ECS is 10% slower due to the need to rearrange patterns^25^, and conventional compressive sensing is slower due to the increased computational overhead.

These results show that even in this low resolution regime conventional compressed sensing still performs well for low sampling ratios (i.e. high compression), though with a penalty of long reconstruction times. The Russian Dolls performance for low sampling ratios seems to be excellent, and indeed it achieves the lowest RMSE of all methods. By contrast ECS can contain finer details and does not incur long reconstruction time penalties, however, practical implementations require *a priori* knowledge of the scene which in practice comes from the previous frames, resulting in errors in scenes with motion.

**Figure 4:**
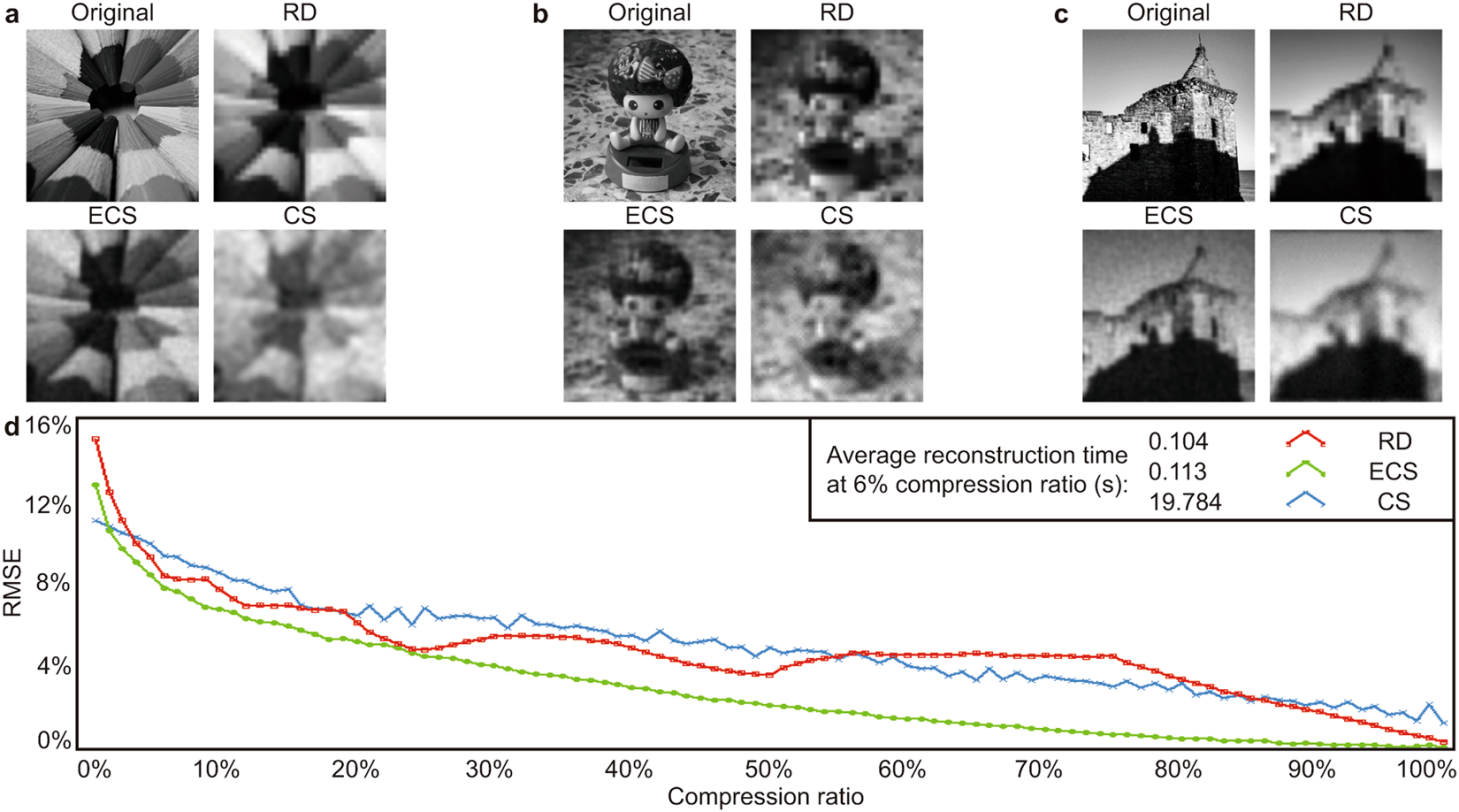
General scene reconstruction comparison. (**a**–**c**) Examples of general scene (Original), the reconstructed images using ‘Russian Dolls’ approach (RD), evolutionary compressive sensing (ECS), and conventional compressive sensing (CS). (**d**) Comparison based on relative error per pixel as a function of sampling ratio. Average reconstruction times at 6% sampling ratio for three approadches are given as well.

In the second simulation, the set contains 35 images (examples labelled as ‘Original’ in Fig. 4a–c), which aims to simulate imaging of general scenes and some examples for a 6% sampling ratio are shown in Fig. 4a–c. Figure 4d illustrates the comparison results for this set. The ‘Russian Dolls’ result still shows the same characteristic at the sampling ratios of 50%, 25%, 12%, 6% and 3%, where the relative errors exhibit local minima. However, in this simulation, the performance of the ‘Russian Dolls’ approach is diminished due to the absence of a uniform dark background, as this maximises the effect of a perfect reconstruction. The evolutionary compressive sensing outperforms the other two approaches, however this is in the limit of optimal *a priori* information (we know all values of *S*_*i*_) and any real application does not have this luxury. Conventional compressive sensing performs better only when the sampling ratio is small and still requires computationally intensive reconstruction.

Besides the observations above, although the relative error per pixel is an overall criterion assessing how similar the reconstructed image is to the original one, it can be inconsistent with visual impression. We also note that the conventional compressive sensing performed in this work is only a representative method within a broad field. We emphasise that these results hold for the specific case of moderate resolution, which is chosen to be compatible with video rate image acquisition. We have confirmed that in this moderate resolution regime traditional compressed sensing does not have the impact seen for high resolution applications while still requiring long reconstruction times. Our ‘Russian Dolls’ technique provides similar RMSE results to the other methods, we believe that this method can be useful due to its speed and lack of reliance on *a priori* information. Ultimately, the specific imaging application will inform which technique has the best performance and we believe that this ‘Russian Dolls’ ordering can be useful for low-resolution real-time imaging of moving scenes.

## Discussion

In this work, we proposed an optimized order of the Hadamard basis for use in compressive single-pixel imaging applications. The Russian Dolls ordering utilizes the sparsity of natural scenes, similar to transform coding^30, 31^. Our numerical simulations demonstrate that this ‘Russian Dolls’ order of the Hadamard basis can yield a similar image quality compared to conventional or evolutionary compressive sensing but with minimal computational resource, and is not limited to binary images^32^. In the case of a properly chosen sampling ratio and imaging a single object on a uniform background, this ‘Russian Dolls’ approach outperforms the other methods with regards to SNR and image reconstruction, but suffers from reduced detail. Furthermore, without a computational overhead, the ‘Russian Dolls’ method reconstructs images significantly faster than conventional compressed sensing. Therefore, this method can be utilised to improve real-time performance in single-pixel video applications, particularly where *a priori* estimate of the scene is unavailable or unreliable.

## Data Availability

The datasets generated during and/or analysed during the current study are available from the corresponding author on reasonable request.
